# Correlating Fluorescence and High-Resolution Scanning Electron Microscopy (HRSEM) for the study of GABA_A_ receptor clustering induced by inhibitory synaptic plasticity

**DOI:** 10.1038/s41598-017-14210-5

**Published:** 2017-10-23

**Authors:** Marta Orlando, Tiziana Ravasenga, Enrica Maria Petrini, Andrea Falqui, Roberto Marotta, Andrea Barberis

**Affiliations:** 1 Neuroscience and Brain Technologies Department, Fondazione Istituto Italiano di Tecnologia, Via Morego 30, 16163 Genoa, Italy; 2 Nabla Lab, Biological and Environmental Sciences and Engineering (BESE) Division, King Abdullah University of Science and Technology (KAUST), 23955-6900 Thuwal, Saudi Arabia; 3 Electron Microscopy Facility, Fondazione Istituto Italiano di Tecnologia, Via Morego 30, 16163 Genoa, Italy; 4 Present Address: Institute of Neurophysiology, Charité-Universitätsmedizin Berlin, Charitéplatz 1, 10117 Berlin, Germany

## Abstract

Both excitatory and inhibitory synaptic contacts display activity dependent dynamic changes in their efficacy that are globally termed synaptic plasticity. Although the molecular mechanisms underlying glutamatergic synaptic plasticity have been extensively investigated and described, those responsible for inhibitory synaptic plasticity are only beginning to be unveiled. In this framework, the ultrastructural changes of the inhibitory synapses during plasticity have been poorly investigated. Here we combined confocal fluorescence microscopy (CFM) with high resolution scanning electron microscopy (HRSEM) to characterize the fine structural rearrangements of post-synaptic GABA_A_ Receptors (GABA_A_Rs) at the nanometric scale during the induction of inhibitory long-term potentiation (iLTP). Additional electron tomography (ET) experiments on immunolabelled hippocampal neurons allowed the visualization of synaptic contacts and confirmed the reorganization of post-synaptic GABA_A_R clusters in response to chemical iLTP inducing protocol. Altogether, these approaches revealed that, following the induction of inhibitory synaptic potentiation, GABA_A_R clusters increase in size and number at the post-synaptic membrane with no other major structural changes of the pre- and post-synaptic elements.

## Introduction

One of the most distinctive features of neuronal networks is their ability to undergo activity dependent plastic changes that modify synaptic strength^1^. While most of the works of the last three decades have mainly focused on excitatory synaptic plasticity, the plasticity of inhibitory connections is far less understood. More recently, it has been demonstrated that inhibitory synapses express several forms of plasticity, indicating that plastic changes of both excitatory and inhibitory synapses play a crucial role in setting the Excitation/Inhibition balance (E/I), a major determinant of network functioning. At inhibitory synapses, several forms of synaptic plasticity have been comprehensively characterized at the pre-synaptic level. Indeed, retrograde messengers released from post-synaptic side (including endocannabinoids, brain-derived neurotrophic factor (BDNF) and nitric oxide (NO)) diffuse back to the presynaptic element, modulating the release machinery and regulating the amount of GABA released in the synaptic cleft^2,3^. In contrast, at the post-synaptic level, the molecular mechanisms responsible for the activity-dependent tuning of inhibitory synaptic responses are only beginning to be revealed^4–7^. An increasing body of evidence has demonstrated that phosphorylation of the GABA_A_Rs by the calcium dependent kinase CaMKII is a key determinant for the changes in GABA_A_Rs functioning^8^ and localization at synapses^9^. Along the same lines, diverse post-translational modifications of the inhibitory scaffold protein gephyrin modulate the aggregation state of synaptic gephyrin, thus regulating the GABA_A_R anchoring/clustering at synaptic sites^10–12^. Previous work by our group showed that, during potentiation of inhibitory synapses, gephyrin is redistributed from the extrasynaptic to the synaptic area upon CaMKII phosphorylation of GABA_A_R with consequent immobilization and accumulation of GABA_A_R at synaptic sites^13,14^.

Differently from inhibitory synapses, which are defined symmetric, asymmetric excitatory synapses are located on dendritic varicosities called “spines”. By partially segregating the post-synaptic *milieu*, dendritic spines play a pivotal role in the synapse functioning both in basal conditions and during synaptic plasticity^15^. Indeed, activity-dependent structural remodelling of dendritic spines has been observed during synaptic plasticity by super-resolution fluorescence microscopy and electron microscopy^16–19^. While a large number of studies addressed the structural plasticity of glutamatergic spines, the fine changes in the morphology of GABAergic synapses during synaptic plasticity have been far less characterized. Inhibitory synapses do not have a clear post-synaptic density and their structural remodelling has not been studied as thoroughly as that of excitatory post-synaptic sites. Nevertheless a proteomic study has recently identified a far more complex protein network at inhibitory postsynaptic sites than previously thought^20^. The aim of the present work is to reveal at nanometric resolution the modifications of inhibitory GABAergic synapses following the expression of a post-synaptic form of inhibitory long-term potentiation (iLTP). In particular, by correlative light-high resolution scanning electron microscopy (CL-HRSEM) and electron tomography (ET) we examined the degree of synaptic clustering of surface GABA_A_Rs and the properties of the pre- and post-synaptic membranes before and after the delivery of the plasticity-inducing protocol. In detail we combined confocal fluorescence microscopy (CFM) with high resolution scanning electron microscopy (HRSEM) on the same region of interest^21–33^ to immuno-localize GABA_A_Rs epitopes at the surface of the neuronal membrane, and we performed electron tomography (ET) to explore the intracellular ultrastructure in 3D at nanometric scale^34–38^.

To acquire information on the distribution of GABA_A_Rs at nanometre scale, neurons grown on glass coverslips with a photo-etched finder grid were labelled with primary antibodies directed against the GABA_A_R α1 subunit (GABA_A_Rα1) and with secondary antibodies conjugated with FluoroNanogold^TM^ (fluorescein isothiocyanate combined to 1.4 nm gold nanoseeds, Nanoprobes) after iLTP induction or a control treatment. The fluorescence of the FluoroNanogold^TM^ was first imaged by confocal microscopy (CFM) in order to get a first map of GABA_A_R clusters and localize neurons of interest on the gridded coverslips, with the typical CFM lateral resolution. Samples were then processed for HRSEM after a gold enhancement reaction to increase the size of gold nanoseeds (see Materials and Methods for details). The images of the detected backscattered electron (BSE) and the secondary electron (SE) signals were then simultaneously acquired in order to localize gold nanoparticles, (which provide high compositional contrast in BSE images) while imaging the fine surface morphology by SE signal. In another set of experiments we performed ET on hippocampal neurons immuno-labelled for GABA_A_Rα1. This allowed us to precisely localize gold particle clusters on post-synaptic membranes facing GABAergic presynaptic boutons in control conditions and during iLTP. The aforementioned approaches revealed an increased clustering of GABA_A_Rα1 receptors at GABAergic synapses upon the induction of inhibitory synaptic potentiation. Importantly, the expression of such inhibitory plasticity occurred in the absence of major structural rearrangements of the pre- and post-synaptic membranes. Those results indicate that, upon the conditions examined here, the expression of inhibitory synaptic potentiation does not require structural plasticity, leaving the modulation of GABA_A_Rα1 receptor trafficking and lateral diffusion as the main determinants of iLTP, differently from what happens in excitatory synapses.

## Results

### GABA_A_Rα1 clusters are detectable with high resolution at the scanning electron microscope

**Figure 1:**
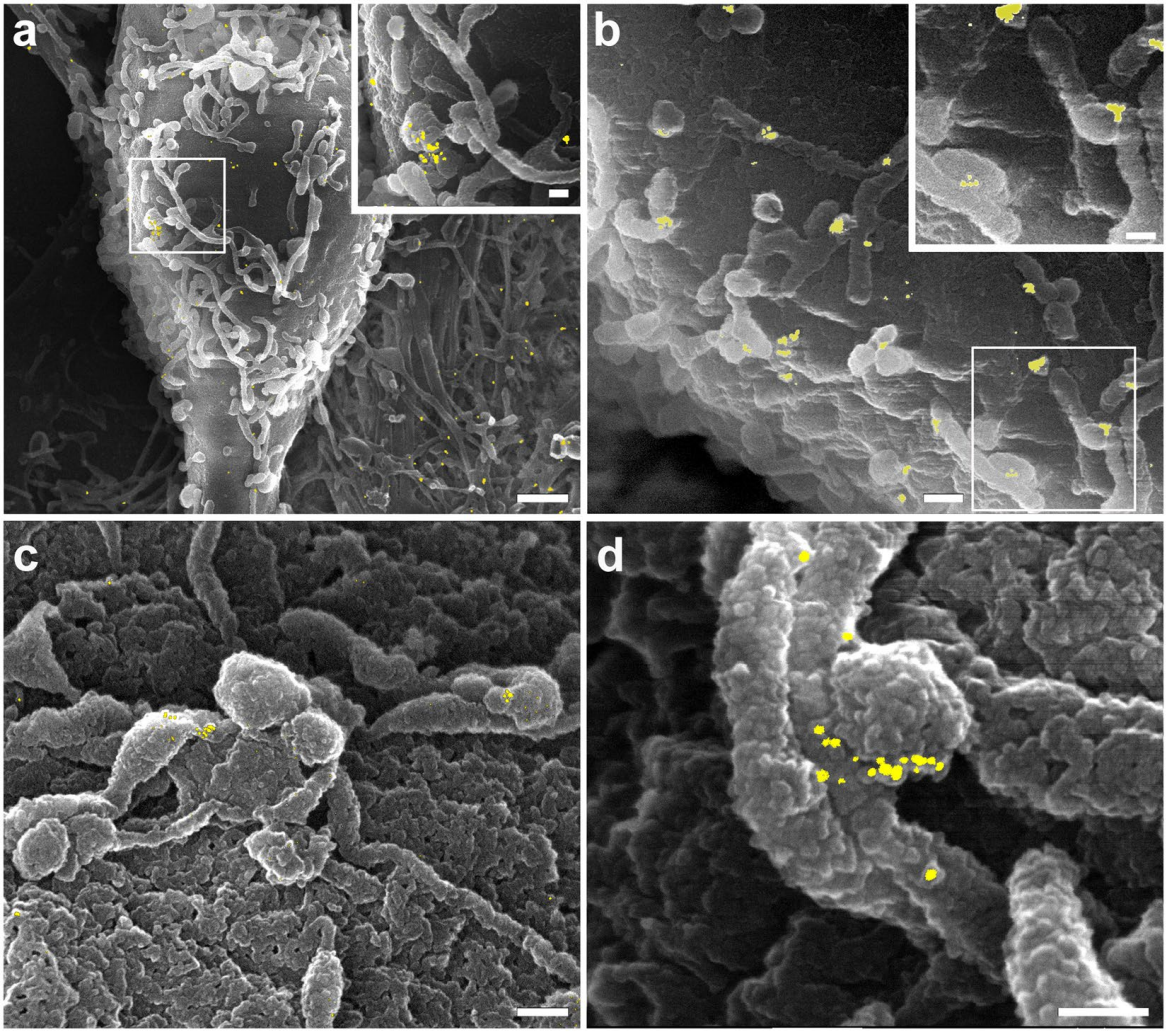
High resolution immuno-scanning electron microscopy (HRSEM) images of GABA_A_Rα1 in primary mouse hippocampal neurons labelled using FluoroNanogold^TM^ or 10 nm colloidal gold particle-conjugated secondary antibody. The BSE signal (pseudo-coloured in yellow) is superimposed on the SE images. (**a**): cell body of a neuron at low magnification. Scale bar: 1 µm. Several neurites are present on the cell body plasma membrane. Inset: high magnification of the boxed region in a. Scale bar: 0.2 µm; (**b**): detail showing the presence of gold clusters at contact regions between processes. Scale bar: 1 µm Inset: high magnification of the boxed region in b. Scale bar: 0.1 µm; (**c**): a group of neurites on the cell body plasma membrane. Note the presence of gold clusters at contact regions between swollen neurite terminals. Scale bar: 0.2 µm; (**d**): contact region between neurites imaged at high magnification. Note the presence of gold clusters at the swollen neurite terminal. Scale bar: 0.1 µm. Contact regions between processes are *bona fide* synaptic contacts.

BSE analysis on primary hippocampal neuron cultures immunolabelled with an anti-GABA_A_R α1 subunit (GABA_A_Rα1) primary antibody followed FluoroNanogold^TM^ Fab secondary antibody revealed the presence of GABA_A_Rα1 gold clusters of variable size (due to gold-enhancement reaction) on both soma and neurites (Fig. 1 and Fig. S1). At higher magnification, gold clusters appeared to be located on the plasma membrane in close proximity to neuritic processes covering the neuron cell body (Fig. 1a,b and Fig. S1a,b). Gold clusters were also found at contact regions between processes i.e., in *bona fide* synaptic contacts (Fig. 1b). A similar gold clusters distribution was observed in a parallel set of experiments in which the same immunolabelling of GABA_A_Rα1 on primary cultured hippocampal neurons (with the same primary antibody) was revealed with a 10 nm colloidal gold particle-conjugated secondary antibody, instead of the FluoroNanogold^TM^ (Fig. 1c,d and Fig. S1c,d). Importantly, control experiments performed in the absence of primary antibodies showed no gold clusters (Fig. S2).

### Fluorescence microscopy images of live labelled receptor clusters can be correlated with high resolution scanning electron microscopy images (HR-SEM)

**Figure 2:**
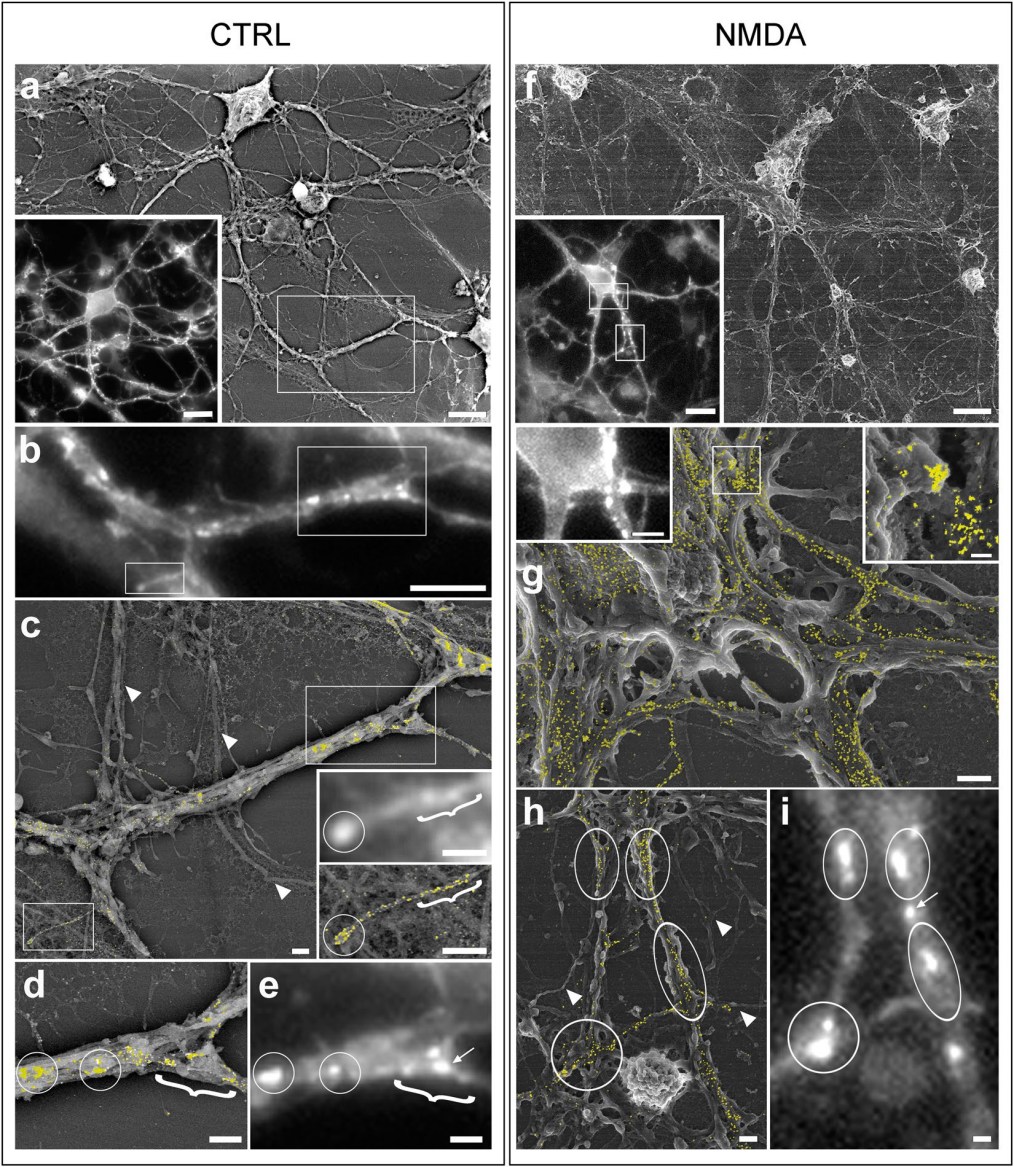
Correlative light-high resolution scanning electron microscopy (CL-HRSEM) localization of GABA_A_Rα1 in control (CTRL, a–e) and iLTP (NMDA, f–i) primary hippocampal neurons growing on photo-etched coverslips. The BSE signal (pseudo-coloured in yellow) is superimposed on the grey-scale SE images. (**a**): low magnification HRSEM images of a CTRL neuron immunolabelled for GABA_A_Rα1. Inset: same neurons imaged by CFM. (**b**): CFM image of part of the neurite bundle boxed in a. Note the presence of GABA_A_Rα1 clusters (bright spots) along the neurite. The boxed regions are magnified in c (insets) and in d–e. (**c**): HRSEM image showing the same region boxed in a. The yellow spots on the neurite bundle are GABA_A_Rα1 receptor clusters. The double inset shows higher magnifications of the single neurite boxed in (c) (bottom left) imaged respectively at the CFM (above) and at the HRSEM (below). The arrowheads point to neurites without gold nanoparticles; (**d**): HRSEM image of the bundle of neurites boxed in c (upper right); (**e**): CFM image of the same bundle of neurites imaged in d. The arrow points to a fluorescent spot not observed in d; (**f**): low magnification HRSEM images of a NMDA stimulated neurons immunolabelled for GABA_A_Rα1. Inset: the same neurons imaged by CFM; (**g**): HRSEM image showing a portion of the cell body of the neuron imaged in f (upper Inset). Left inset: the same region imaged at the CFM. Right Inset: HRSEM higher magnification of the region boxed in g. (**h**): HRSEM image of the region boxed in f (bottom Inset). The arrowheads point to neurites without gold nanoparticles; (**i**), CFM image of the same region imaged in h. The arrow points to a fluorescent spot not observed in h. Circles and brackets point to the same sub-regions. Scale bars are 10 µm in a, a inset, f, f inset; 5 µm in b and g right inset; 1 µm in c–e and g–i; 0.2 µm in g right inset.

**Figure 3:**
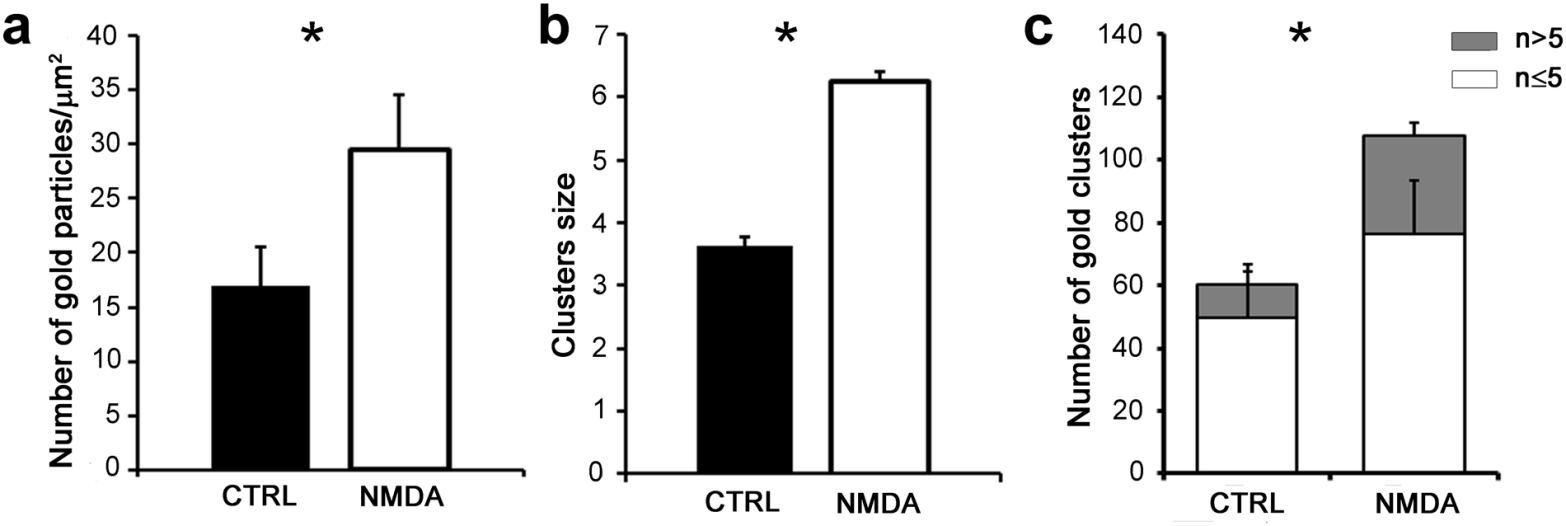
Subcellular GABA_A_Rα1 receptor distribution on soma and neurites in CTRL and NMDA stimulated hippocampal neurons. (**a**): bar plot showing gold clusters density in CTRL and NMDA stimulated neurons; (**b**): bar plot showing clusters size (number of gold particles/cluster) in CTRL and NMDA stimulated neurons; (**c**): bar plot showing the number of clusters formed respectively by n ≤ 5 and n > 5 gold particles on soma and neurites in CTRL and NMDA stimulated hippocampal neurons. * Indicates significant differences (*p < 0.05, Student’s t-test). Values are mean ± s.e.m.

Confocal fluorescence microscopy (CFM) imaging was conducted to obtain a map of GABA_A_Rα1 clusters and to localize neurons of interest on gridded coverslips. This analysis revealed the presence of GABA_A_Rα1 bright puncta on soma and neurites in both control and iLTP neurons (Fig. 2). In line with our previous findings^14^, the fluorescence intensity of GABA_A_Rα1 signal was larger in NMDA treated neurons as compared to controls. When the same samples were observed by SEM at low magnification, BSE analysis showed intensely labelled regions finely spotted along the neuronal membrane (Fig. 2a,f). As a side note, such signal was absent in negative controls, i.e. omitting the primary antibody (not shown). At higher magnification, the bright BSE immune-positive patches appeared as clusters of gold particles, corresponding to individual signal spots on soma and neurites that correlated with the post-synaptic clusters of GABA_A_Rα1 imaged in CFM (see Fig. 2b–e for CTRL and 2g–i for NMDA). As expected, no spotted BSE signal was observed in neuronal regions with no fluorescence (compare Fig. 2d with e and Fig. 2h with i). Along the same lines, neurites exhibiting low density BSE signal corresponded to regions with low and diffused fluorescence intensity (compare Fig. 2c right insets and 2 g insets). Although many of the bright immune-positive fluorescence patches matched with individual densely labelled BSE regions, some of the single bright patches corresponded to more than one cluster of gold nanoparticles (see encircled regions in Fig. 2c right insets and Fig. 2d,e). In other cases, single bright patches corresponded to regions where the nanoparticles were present at high density but without forming clusters (see encircled regions in Fig. 2h and i). Only in 7.27% of cases (4/55) we observed fluorescent bright patches with missing or loosely correlated nanoparticles (see also the regions indicated by an arrow in Fig. 2d,e and in Fig. 2h,i). Next, to quantitatively estimate gold clusters in control and iLTP neurons, we analysed their density (number of gold particles/µm^2^), their size (average number of gold particles/cluster) and the number of clusters formed respectively by n ≤ 5 and n > 5 gold particles. In agreement with the CFM results, following plasticity induction with NMDA treatment, the average density of gold clusters significantly increased as compared to controls (from 16.9 ± 3.6 clusters/µm^2^ in CTRL to 29.4 ± 5.1 clusters/µm^2^ in iLTP, Fig. 3a and S_Table 1). Moreover, in NMDA stimulated neurons, we measured a significant increase of ~40% in the average size of the gold clusters with respect to controls (from 3.6 ± 0.2 gold particles/cluster in CTRL to 6.2 ± 1.2 in NMDA, Fig. 3b and S_Table 1). To further investigate the GABA_A_Rα1 organization at the post-synaptic terminal, we grouped the observed clusters on the basis of the number of gold particles per cluster (n = number of gold particles; 1^st^ class: n ≤ 5; 2^nd^ class: n > 5). In the NMDA treated neurons we measured a general increase in the number of gold clusters belonging to each class with respect to controls, especially the 2^nd^ class (>5 gold particles) that nearly tripled (from 10.4 ± 4.2 clusters in CTRL to 31.07 ± 7.1 clusters in NMDA treated neurons, Fig. 3c and Table S1). Overall those results indicate that the induction of inhibitory synaptic potentiation with NMDA in hippocampal neurons elicits a marked increase of GABA_A_Rα1 clusters, especially those exhibiting a larger number of gold particles.

### Gold labelled clusters are localized on the postsynaptic membrane facing symmetric synaptic *boutons*

**Figure 4:**
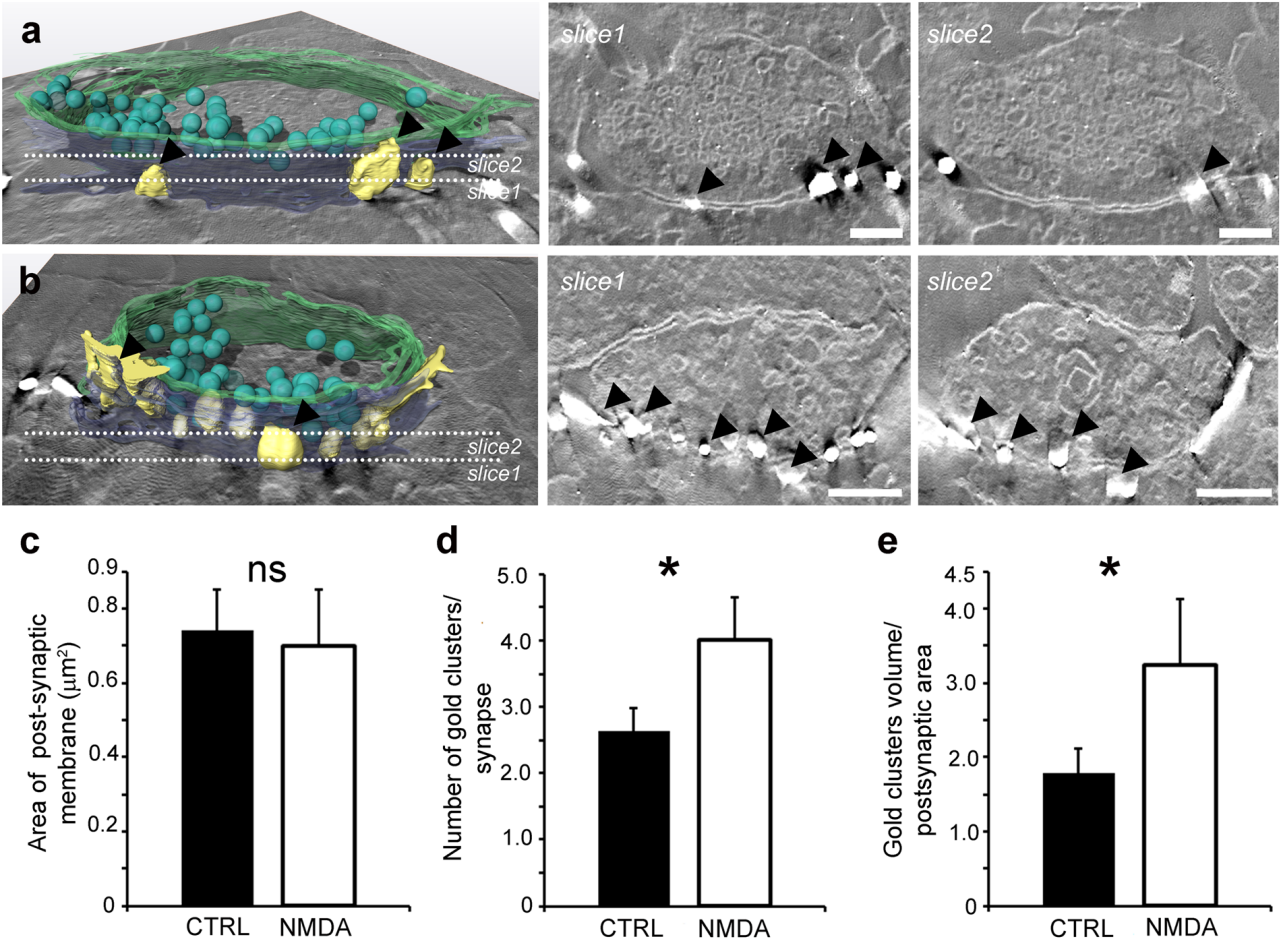
Post-synaptic surface and post-synaptic GABA_A_Rα1 distribution at inhibitory synapses of CTRL and NMDA stimulated hippocampal neurons. (**a**): 3D model of an inhibitory synapse immuno-labelled for the GABA_A_Rα1 in a hippocampal neuron in control conditions (CTRL). Slice1 and slice2 are two tomographic slices through the dotted lines in a. Arrowheads point to gold clusters; (**b**): 3D model of an inhibitory synapse immunolabelled for the GABA_A_Rα1 in a hippocampal neuron after the induction of plasticity by NMDA stimulation. Slice1 and slice2 are two tomographic slices through the dotted lines in b. Arrowheads point to gold clusters; note that in NMDA treated samples, more gold clusters are visible. (**c**): bar plot showing the area of the post-synaptic membrane on GABA_A_Rα1 immunolabelled synapses in both CTRL and NMDA stimulated neurons; (**d**): bar plot showing the number of gold clusters/synapse in CTRL and NMDA stimulated neurons; (**e**): bar plot showing the gold clusters volume normalized over the post-synaptic area in CTRL and NMDA stimulated neurons. Values are mean ± s.e.m. * Indicates significant differences (p < 0.01, Student’s t-test); ns = statistically not significant. Colour codes for the 3D models: post-synaptic membrane (blue), pre-synaptic membrane (green), gold clusters (yellow), and neurotransmitter vesicles (cyan). Scale bars: 200 nm.

To further localize and quantify the GABA_A_Rα1 signal specifically at inhibitory synapses we performed ET on primary cultured hippocampal neurons immunolabelled for GABA_A_Rα1 using FluoroNanogold^TM^ Fab secondary antibody followed by gold enhancement. In both controls and NMDA-treated neurons we observed the presence of gold clusters along the post-synaptic membrane facing the pre-synaptic terminal of symmetrical synapses (Fig. 4 and Movie S1 and S2). To assess whether inhibitory synapses underwent ultrastructural remodelling following the induction of synaptic plasticity, we measured the surface of the post-synaptic membrane facing the pre-synaptic terminal in both controls and NMDA treated neurons. This analysis revealed no differences between synapses in control and stimulated neurons (0.74 μm^2^ ± 0.11, n = 14 in CTRL and 0.70 µm^2^ ± 0.15, n = 12 in NMDA neurons, Fig. 4c and Table S2). We then characterized the fine organization of the gold clusters decorating the post-synaptic membrane in both controls and NMDA stimulated neurons. Congruently with the HRSEM results, the average number of gold clusters at the post-synaptic membrane increased significantly in the NMDA stimulated neurons with respect to the controls (from 2.6 ± 0.3, n = 14 in CTRL to 4.0 ± 0.6, n = 13 in NMDA treated neurons, Fig. 4a,b,d and Table S2). Such increase of gold cluster number was paralleled by a significant enhancement of the average volume of gold clusters/post-synaptic area, that nearly doubled in NMDA stimulated neurons with respect to controls (from 1.8 ± 0.3 µm, n = 14 in CTRL to 3.3 ± 0.9 µm, n = 12 in NMDA treated neurons, Fig. 4a,b,e and Table S2). Altogether, these results indicate that, compared to control conditions, inhibitory synaptic potentiation is associated with more GABA_A_Rs clusters composed of an increased number of receptors in the absence of alterations in the structural features of symmetric synapses. Thus, both GABA_A_R trafficking and redistribution via lateral diffusion contribute to inhibitory synaptic plasticity allowing fast and reliable strengthening of the inhibitory signal with no major remodelling of the post-synaptic membrane.

## Discussion

By coupling fluorescence microscopy with the high-resolution power of HRSEM, correlative light - high resolution scanning electron microscopy (CL-HRSEM) is an excellent method to study the spatial distribution of target molecules at the cellular surface at nanometric level^39^. Although still undeveloped with respect to fluorescence microscopy combined with transmission electron microscopy (TEM), several recent papers have shown the feasibility of CL-HRSEM by proposing new probes^32^, new substrates for growing cells^40^ and new coating strategies to provide electrical conductivity^28,41,42^. Through the years, immuno-SEM has been used to localize at the nanometre scale the spatial distribution of a variety of surface molecular targets, ranging from phosphorylated histone H3^30^, cell adhesion molecules^43–47^, bacterial and cancer surface^48–50^, collagen fibrils^51^, and cell focal adhesion sites^21^ in a variety of organelles, cell types and tissues. In particular HRSEM in combination with fluorescence microscopy has been used to study a variety of molecular targets on the surface of a multiplicity of cells and tissue types. Two main approaches have been applied: i) the use of exclusively fluorescent probes to mark molecules of interest, and then the superimposition of the fluorescence images with the corresponding high resolution SE image^52–54^ or ii) the use of dual tagged probes^55,56^ or of a mixture of fluorescent and electron-dense probes^28^ to visualize the molecular tags at the HRSEM using BSE, after the overlay of the fluorescent images with the corresponding SE images.

As expected for proteins enriched at synaptic sites, the GABA_A_Rα1 fluorescent labelling we observed on neuron cell bodies and neurites showed a punctate pattern^57^. The same distribution was observed at the HRSEM as intense BSE bright spots. GABA_A_Rα1 clusters were mainly located at GABAergic symmetrical synapses, although a sizable amount of receptor immunolabelling was detected at extrasynaptic sites, i.e. dendritic regions that were not coupled to axonal counterparts^58^. At synapses, GABA_A_Rα1 immunoreactivity was exclusively post-synaptic^59^. The aggregation of GABA_A_Rα1 receptors at symmetric synapses is consistent with their role in fast inhibitory synaptic transmission. In contrast, the GABA_A_Rα1 receptors observed at extrasynaptic areas are those that dynamically exchange between synapses and exocytosis/endocytosis sites^7^, and may contribute to tonic inhibitory current^58^. The evidence that during iLTP we observed increased GABA_A_ R clustering without major remodelling of the pre- and post-synaptic membranes indicates that inhibitory synaptic potentiation does not require structural plasticity. Hence, the mechanisms underlying iLTP fundamentally differ from those of excitatory LTP, which imply profound modifications of the post-synaptic membrane at spines^60^. These data are in line with recent studies reporting that the synaptic cluster of gephyrin, the main GABA_A_R anchoring protein at inhibitory synapses, showed increased size and complexity following iLTP^13,14^. Thus, we emphasize here that during inhibitory synaptic plasticity, the redistribution of postsynaptic proteins at post-synaptic sites is the main molecular event in the potentiation of GABAergic synapses. The correlative labelling protocol described here could be also applied for wet environmental scanning electron microscopy (wet-ESEM) imaging^61^. This might reduce the spatial resolution as compared to high-vacuum techniques, but would allow investigating the dynamics of receptor redistribution.

## Methods

### Animals

All experiments were carried out in accordance with the guidelines established by the European Communities Council (Directive 2010/63/EU of 22 September 2010) were permitted by the Italian Ministry of Health and followed the rules approved by the Italian Institute of Technology. All animal surgeries were done in agreement with the Italian Ministry of Health Regulation and Authorization and have been approved by the Italian Institute of Technology.

### Primary neuronal cultures, iLTP induction and immunolabelling

Primary cultures of hippocampal neurons were prepared from C57BL/6 J mice at postnatal day 0 (P0) as previously described^14^. Neurons were plated on coverslips with a photo-etched counting grid (BELLCO GLASS INC.) and kept in Neurobasal medium in incubator at 37 °C with 5% CO_2_. To induce inhibitory synaptic potentiation, at 13–16 days *in vitro* (DIV) neurons were treated for 2 minutes with NMDA 20 μM (Sigma, Italy) and CNQX 10 μM (Tocris, Italy) dissolved in an extracellular recording solution (containing in mM: 145 NaCl, 2 KCl, 2 CaCl_2_, 2 MgCl_2_, 10 glucose, and 10 HEPES, pH 7.4) and then washed and incubated in the extracellular solution to allow recovery and expression of iLTP^14^. During the recovery period, 12 minutes after the end of the NMDA treatment, live immunolabelling of GABA_A_Rα1 subunits was performed, namely cells were incubated 13 minutes in a solution containing primary antibody against GABA_A_Rα1 (Alomone Labs, Israel) diluted 1:30 in 0.5% bovine serum albumin (BSA), 350 mM sucrose in PBS, followed by 13 minutes incubation with gold-conjugated secondary antibody. A first set of experiments was performed using secondary antibody conjugated to 10 nm gold particles (Aurion, diluted 1:25 in 0.5% BSA, 350 mM sucrose in PBS). To reduce the size of our probe and to directly correlate CFM and electron microscopy we then used FluoroNanogold^TM^ (Nanoprobes, diluted 1:25 in 0.5% BSA, 350 mM sucrose in PBS) since it exhibits both an Alexa 488 fluorochrome and a colloidal nanogold particle (1.4 nm) conjugated to an IgG Fab fragment. In control samples, NMDA and CNQX were omitted. To check for non-specific binding of the secondary antibodies, in control experiments the primary antibody was omitted.

### Correlative light high resolution scanning electron microscopy (CL-HRSEM)

Live confocal and transmitted fluorescence images where acquired with a Nikon Live scan SFC inverted microscope at various magnifications (i.e. 20x, 40x, 60x). Lower magnifications were used to localize neurons on the reference photo-etched grid, while the 60x objective was used to acquire fluorescence images of GABA_A_Rα1 clusters. Subsequently, the same samples were prepared for SEM analysis. Cells were fixed for 1 hour in 1.2% glutaraldehyde in PBS followed by enhancement of the FluoroNanogold^TM^ particles size with a gold enhancement kit (GoldEnhance, Nanoprobes) until reaching a final size of about 10 nm to allow their detection by backscattered electrons (BSE) signal in HRSEM. Neurons were then post-fixed in 1% osmium tetroxide in 0.1 M sodium cacodylate (pH 7.4), dehydrated in a graded series of cold ethanol followed by complete dehydration in hexamethyldesilanzane (HMDS). Complete evaporation of HMDS was obtained overnight and the following days samples were sputter-coated with a thin (5 nm) layer of chromium to allow surface conductivity. Samples were then transferred in the SEM right after the sputter coating to limit the fast chromium oxidation. HRSEM imaging was performed by a JEOL JSM 7500 F microscope equipped with a cold field emission gun and working at an acceleration voltage of 15 kV. In-lens detector and in-chamber multi-quadrant annular retractable solid state detector collected the secondary electrons and backscattered electrons signals, respectively. Both SE and BSE signals were simultaneously recorded, keeping the samples at a fixed working distance of 8 mm.

### Electron Tomography

For ET 3D imaging neurons that were live-labelled for GABA_A_Rα1 (see above) were then fixed for 1 hr at room temperature in 1.2% glutaraldehyde in 66 mM sodium cacodylate buffer (pH 7.4) and processed for conventional resin embedding as described by Giacomini and colleagues^36^. Semi-thin sections of about 350 nm of the embedded cell monolayer were cut with an ultramicrotome (Leica UC6, Austria) equipped with a diamond knife (Histo diamond knife 45°, Diatome) and collected on Formvar/carbon coated copper slot grids. Electron tomography was performed in scanning transmission electron microscopy (STEM) working in high angular annular dark field (HAADF) geometry, using a FEI Tecnai F20 transmission electron microscope operating at 200 kV and equipped with a Schottky field-emission gun. Such geometry was chosen in order to rule out any diffraction contrast contribution to the collected images. The tomographic series where collected from 350 nm thick sections tilted over ± 60 degrees with the following tilt scheme: 1 degree angular step for tilt angle higher/lower than ± 30 degrees, and 2 degrees angular step between ± 30 degrees. The images were acquired with magnification ranging from 40.000 and 56.000, corresponding to a pixel size comprised between 2.59 and 1.85 nm respectively. Computation of tomograms was done by weighted back projection (WBP)^62^ with IMOD 4.8 software package^63^. Both segmentation and 3D visualization were performed using the Amira package (FEI Visualization Science Group, Bordeaux, France).

### EM quantitative analysis

Image analysis on HRSEM data has been performed using ImageJ 1.50i^64^. The gold clusters density has been calculated using the “analyse particles” function of ImageJ on a total of 54 HRSEM BSE and SE images of iLTP and control samples (CTRL: n = 20 neuron images collected in 3 different experiments; NMDA: n = 44 neuron images collected in 4 different experiments). The clusters size (number of gold particles/cluster) has been calculated on a total of 2244 randomly picked gold clusters from HRSEM images coming from different experiments (CTRL: n = 584 from 2 experiments; NMDA: n = 1660 from 3 experiments). Moreover more than 4800 randomly picked gold clusters (CTRL: n = 662 from 2 experiments; NMDA: n = 4169 from 3 experiments) were arbitrary subdivided in classes formed by n ≤ 5 and n > 5 gold particles. The analysis on the tomographic 3D models of inhibitory synapses in both control and iLTP hippocampal neurons have been performed using the Amira package (FEI Visualization Science Group, Bordeaux, France). The post-synaptic terminal surface and the number and volume of gold clusters at the inhibitory synapse have been extracted using the MaterialStatistics module as implemented in Amira from a total of 26 tomographic reconstructions from four different immuno-EM experiments (CTRL: n = 14; and 12 for respectively CTRL and iLTP). To analyse CLM puncta which did not show nanogold clusters at the SEM level, we correlated CLM and HRSEM areas with the help of the finder photo-etched grid and clusters were manually quantified using ImageJ 1.50i^64^. Statistical significance of normally distributed datasets was tested using a two tailed Student’s t-test. A p < 0.05 level was considered as statistically significant; to test for normality the Kolmogorov–Smirnov was performed prior to statistical analysis.

## Electronic supplementary material

Supplementary Information

Movie S1

Movie S2
