# Supplementary material for: Correlating Fluorescence and High-Resolution Scanning Electron Microscopy (HRSEM) for the study of GABAA receptor clustering induced by inhibitory synaptic plasticity
Source: Sci Rep. 2017 Oct 23;7:13768. doi: 10.1038/s41598-017-14210-5 (PMC5653763; doi:10.1038/s41598-017-14210-5)
Supplement: Supplementary file 1 — Supplementary Information [file 41598_2017_14210_MOESM1_ESM.pdf]

Correlating Fluorescence and High-Resolution Scanning Electron Microscopy (HRSEM) for the study of $GABA_A$ receptor clustering induced by inhibitory synaptic plasticity

Marta Orlando[1±§], Tiziana Ravasenga[1±], Enrica Maria Petrini[1±], Andrea Falqui[2], Roberto Marotta[3#] and Andrea Barberis[1*#]

[1]Neuroscience and Brain Technologies Department, Fondazione Istituto Italiano di Tecnologia, Via Morego 30, Genoa, Italy
[2] Nabla Lab, Biological and Environmental Sciences and Engineering (BESE) Division, King Abdullah University of Science and Technology (KAUST), 23955-6900 Thuwal, Saudi Arabia
[3] Electron Microscopy Facility, Fondazione Istituto Italiano di Tecnologia, Via Morego 30, 16163 Genoa, Italy

[±] equal contribution

[§] present address: Institute of Neurophysiology, Charité-Universitätsmedizin Berlin, Charitéplatz 1, 10117, Berlin, Germany

[#] co-last authors

* Correspondence should be addressed to A.B. (email: andrea.barberis@iit.it)

Keywords: immuno-EM, SEM, inhibitory synapse, $GABA_A$ receptor, Electron Tomography, CL-HRSEM, synaptic plasticity

Supplementary figures

Fig. S1

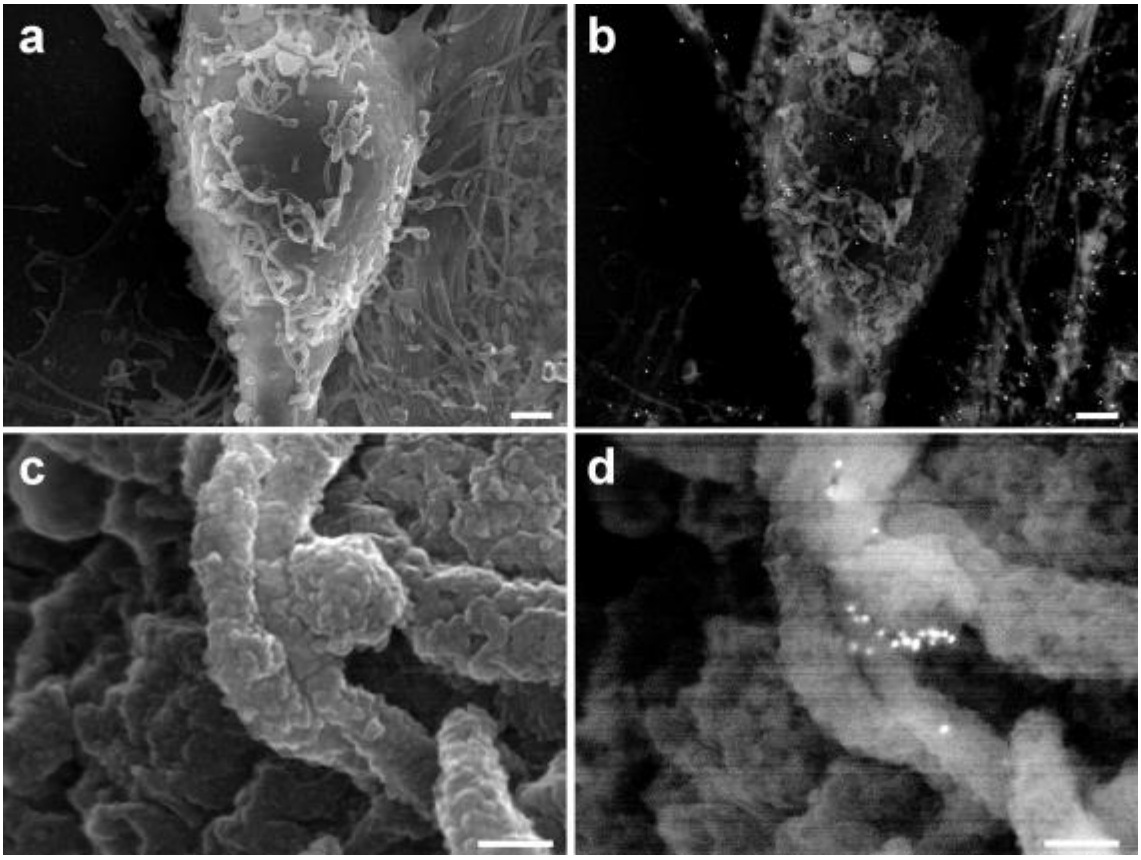

Fig. S1. SE coupled with BSE images of GABA$_A$Rα1 immunolabeled primary mouse hippocampal neurons using FluoroNanogold^{TM} (a and b) and 10 nm colloidal gold particle-conjugated secondary antibody (c and d). a: low magnification SE image of a neuron cell body. b: BSE image of the same field of view shown in a. The white spots are the enhanced gold nanoparticles. c: high magnification SE image of a contact region between neuritis, i.e. *bona fide* synaptic contact. d: BSE image of the same field of view shown in d. The white spots are the 10 nm gold nanoparticles. Scale bars:1 µm in a and b; 0.1 µm in c and d.

Fig. S2

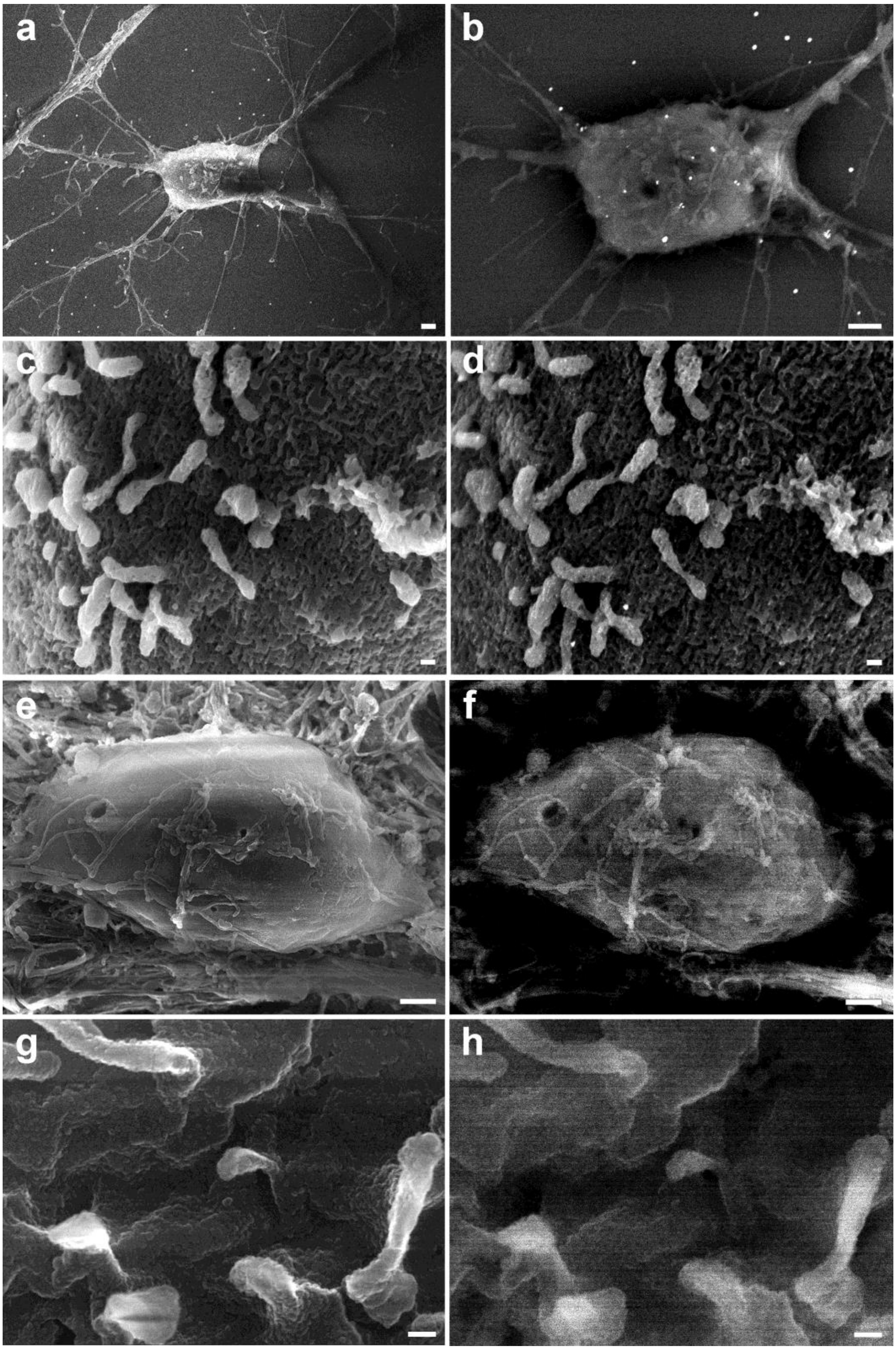

Fig. S2. SE coupled with BSE images of negative control primary mouse hippocampal neurons using FluoroNanogold^{TM} followed by gold enhancement (a-d) and 10 nm colloidal gold particle-conjugated secondary antibody (e-h). a: low magnification SE image of a neuron cell body. b: BSE image of the same field of view shown in a. The large white spots on the coverslip and on the neuron are FluoroNanogoldTM non-specific labelling. c: high magnification SE image of a cell body region. d: BSE image of the same field of view shown in c. The small white spots on the background are non-specific labelling produced by the gold enhancement reaction. The small white spots are the 10 nm gold nanoparticles. e: low magnification SE image of a neuron cell body. f: BSE image of the same field of view shown in e. f: high magnification SE image of a cell body region. d: BSE image of the same field of view shown in d. Scale bars:1 µm in a, b and e, f; 0.1 µm in c, d and g, h.

Table S1. Average cluster density, average cluster size (n gold particles/cluster) and average number of clusters formed by n≤5 and n>5 gold particles on the cell membrane of cell bodies and neurites of CTRL and NMDA stimulated hippocampal neurons. n_i=image number; n_c=cluster number. Values are average ± s.e.m.

	Cluster average density ± s.e.m. (n/ μm^2)		*Clusters size ± s.e.m.*		*Average number of clusters formed by n gold particles ± s.e.m.*			
						n ≤5	***n>5***	***Total***
CTRL neurons	16.9 ± 3.6	n_i=19	3.6 ± 0.2	n_c=584		49.8 ± 17.1	10.4 ± 4.2	n_c=662
NMDA neurons	29.4 ± 5.1	n_i=44	6.2 ± 1.2	n_c=1660		76.5 ± 14.4	31.0 ± 7.1	n_c=4169

Table S2. Average post-synaptic membrane surface, average number of gold clusters *per* synapse and average gold cluster volume *per* synapse and post-synaptic area on CTRL and NMDA mice hippocampal neurons measured on tomograms. Values are average ± s.e.m.

	Average post-synaptic membrane surface (μm^2) ± s.e.m.		*Average number of gold clusters/synapse ± s.e.m.*		*Average gold cluster volume/synapse (µm3) ± s.e.m.*		*Average gold cluster volume/post synaptic area (µm) ± s.e.m.*	
CTRL neurons	0.74 ± 0.11	n=14	2.6 ± 0.3	n=14	0.001 ± 0.0002	n=14	1.8 ± 0.3	n=14
NMDA neurons	0.70 ± 0.15	n=12	4.0 ± 0.6	n=12	0.002± 0.0005	n=12	3.2 ± 0.9	n=12

Movie S1. HAADF STEM WBP tomogram and relative 3D model of an inhibitory synapse immunolabeled for the $GABA_A$Rα1 in a CTRL hippocampal neuron as shown in Figure 4A.

Movie S2. HAADF STEM WBP tomogram and relative 3D model of an inhibitory synapse immunolabeled for the $GABA_A$Rα1 in a NMDA stimulated hippocampal neuron as shown in Figure 4B.
